# Humor in professional coaching: a literature review and research agenda

**DOI:** 10.3389/fpsyg.2024.1288104

**Published:** 2024-07-04

**Authors:** Adélka Vendl, Cristina Alvarado-Alvarez, Martin Euwema

**Affiliations:** ^1^Department of Occupational and Organizational Psychology, KU Leuven, Leuven, Belgium; ^2^Universitat Autònoma de Barcelona, Barcelona, Spain

**Keywords:** coaching, humor, working alliance, coaching effectiveness, positive psychology

## Abstract

**Introduction:**

Extensive research has explored the incorporation of humor in therapy, revealing its potential positive effects on clients’ mental well-being and personal growth. However, limited research exists on how coaching could benefit from humor as an intervention and how its utilization impacts the interaction processes and outcomes for both the coachee and coach. Therefore, our research focuses on the use and effects of spontaneous humor within professional dialogues. This paper aims to extract insights from academic literature on humor in adjacent fields and apply these insights to the context of coaching.

**Methods:**

This paper offers implications for coaching theory and practice, alongside a proposed research agenda. The initial phase involves analyzing reviews on humor in professional contexts, and coaching. Secondly, following the PRISMA guidelines for review, we identified 13 empirical studies, which address the role of humor in counseling, psychotherapy, and mentoring.

**Results and discussion:**

Our findings suggest that humor serves as a valuable tool for establishing and deepening the working alliance, fostering adaptive coping mechanisms in clients, and enhancing the cognitive and behavioral process. Moreover, humor is shown to be advantageous for professionals in navigating challenging client relationships. These findings hold significance for the realm of coaching practice as well. In light of these insights, we propose the integration of humor use in education toolkits for coaching professionals.

## 1 Introduction

Humor is one of the most effective forms of communication, yet it is often overlooked in professional discourse. Over the last few decades, humor has gained increasing attention in mental health literature, spanning fields such as psychotherapy, counseling, education, Human Resource Development (HRD), and family therapy. It serves as a resilience force amidst life stress, creating an environment conducive to non-defensive exploration and fostering social interactions (Valentine and Gabbard, 2014). While professional sources (Beermann and Samson, 2012; Schütz and Kaul, 2022) and gray literature such as dissertations (Dornaus, 2016; Liebman, 2022), underscore the significance of humor in coaching, academic research remains limited. Given humor’s demonstrable efficacy in enhancing mental health, extending this understanding to coaching is imperative. A survey of executive coaching practices highlights humor as a personal quality for coaches (Bono et al., 2009). Esteemed executive coaches are increasingly recognized for their proficiency in psychological skills, including humor (Wasylyshyn, 2003; Joo, 2005). Narratives such as “Behind closed doors” (de Haan et al., 2013) illustrate the catalytic role of humor in invoking change and aiding learning in coaching practices. In the context of provocative coaching, where the coach challenges clients’ beliefs through paradoxes and exaggeration, humor is consistently employed as a catalyst for change during a session. Humor becomes one of the behavioral techniques for coaches, shaping coaching processes, outcomes, and relationships (Vendl, 2017). Coaching serves as a strategy to support and enhance the learning process, with evidence in the literature suggesting that the use of appropriate humor leads to learning, creating discovery, creativity, and motivation (Savage et al., 2017).

Defining coaching as a professional dialogue aimed at enhancing personal and professional growth through interventions and interactions (Grant and Green, 2018; Wang et al., 2022), coaching manifest in diverse forms, including executive coaching, developmental coaching, mental coaching, life coaching, and workplace coaching. The difference between coaching and psychotherapy (or counseling) is that the latter is more focused on ameliorating issues related to psychological problems emphasizing emotional safety. Coaching is more outcome or goal oriented and coaches need to maintain relationships not only with the client but also with other stakeholders managing confidentiality and contractual issues. The theoretical base of coaching is heavily dependent on neighboring disciplines such as mentoring, counseling, organizational studies, and psychotherapy (Myers and Bachkirova, 2018). Research into humor as a psychological construct in coaching, however, remains absent. Gaps persist in the understanding of how coaching operates and the underlying mechanisms (Wang et al., 2022). Analyzing thoroughly the impact of humor in other professional dialogues like psychotherapy, can contribute to further professionalization of coaching and coaching outcomes. Our study aims to address this gap by integrating concepts from clinical and organizational psychology, as well as mentoring to improve theory development. Furthermore, this study explores spontaneous humor defined as therapist-initiated humor stemming from empathic attunement, distinguishing it from structured interventions (Valentine and Gabbard, 2014). Additionally, we look into client-initiated humor emerging from the interaction.

Humor in mental health serves various positive functions and yields diverse effects, as evidenced in different research domains. One domain focuses on the well-being of the client, where humor contributes by aiding stress management, emotional regulation, and fostering positive emotions (Voss et al., 2020; Wu et al., 2021). When dealing with negative feelings, humor proves effective in mitigating negativity by fostering positive emotions, as demonstrated by functional magnetic resonance imaging studies that probe brain activity (Wu et al., 2021). In theoretical psychotherapy papers, humor is linked to the establishment of a strong working alliance between therapist and client (Meyer, 2007; Beermann and Samson, 2012; Sultanoff, 2013; Thomas et al., 2015; Gladding and Drake Wallace, 2016; Knox et al., 2017). Evidence for this connection is discerned in the positive impact of humor resulting from shared laughter, which enhances social bonding (Silva et al., 2017). Additionally, humor plays a role in boosting creativity, facilitating self-observation, and inducing cognitive and behavioral shifts in clients (Gelkopf and Kreitler, 1996; Irving, 2019). Irving (2019) states that humor nurtures a client’s capacity for playfulness and establishes a secure space to play within therapy. Moreover, humor is identified as a competence of effective therapists; in a study involving interviews with 11 therapists, those who judiciously employed humor demonstrated more positive therapeutic effects (Knox et al., 2017). In addition, the fields of counseling and mentoring acknowledge the mental (relieving tensions and gaining new insights) and physical (activating muscles, increasing blood flow, and releasing endorphins) benefits of humor (Shaughnessy and Wadsworth, 1992; Goldin and Bordan, 1999; Love, 2013; Gladding and Drake Wallace, 2016). Goldin et al. (2006) emphasize humor’s potential to shift a client’s perspective and induce a less serious outlook on problems.

While current humor research in psychotherapy is mainly focused on its valuable impact, it is acknowledged that humor usage carries risks (Berk, 2001; Franzini, 2001; Valentine and Gabbard, 2014; Aultman and Meyers, 2020). These risks include misinterpreting or misunderstanding clients, employing humor inappropriately, or even using humor as a weapon (Franzini, 2001). Using humor could lead to mistrust when the therapist uses it unsuitably by belittling, laughing at, or mimicking the client (Sultanoff, 2013). Addressing these risks remains a challenge in the literature, with limited guidance on minimizing them, aside from advocating cautious humor use or refraining from its use altogether. Few theoretical insights exist on how to effectively teach coaches to use humor, besides suggesting a positive humor style characterized by warmth and compassion (Ortiz, 2000; Franzini, 2001; Valentine and Gabbard, 2014).

Recent review studies on humor in the mental health area, focusing on both adults and children (Haire and MacDonald, 2019; Gonot-Schoupinsky et al., 2020; Sun et al., 2023) or exclusively adults (Brooks et al., 2021; Sarink and García-Montes, 2023), examine diverse forms of humor interventions. These interventions range from clowns and yoga/laughter therapy to comedy training and laughing incentives. Despite the frequent use of qualitative methods in these review studies, they seldom dive into the *interaction* between client and therapist when humor is employed. In everyday life, 70% of the humor between people arises spontaneously during social interaction and is the opposite of canned humor like jokes. It is shaped by the conversation and is difficult to recreate (Martin and Ford, 2018). Since spontaneous humor is so commonly present, one of the insights of this paper is how the coach can use humor intentionally, and interpret and utilize the humorous interactions that arise in unforeseen moments during coaching.

Our research aligns with current coaching research trends, initially focused on coaching effectiveness (Theeboom et al., 2014). Subsequent studies examined individual and organizational outcomes (Jones et al., 2016; Bozer and Jones, 2018). Factors such as coachee characteristics (de Haan, 2019) and useful coaching tools across phases (Richter et al., 2021) have been explored. Recent studies emphasize psychological frameworks for coaching effectiveness (Lai and Palmer, 2019), including the importance of coaching alliance (Lai and Palmer, 2019; Graßmann et al., 2020). Our research aims to deepen understanding of coaching outcomes, such as expressing support, enhancing motivation, or forming a good relationship as Lai and Palmer (2019) suggest.

Despite an abundance of papers and established theories discussing humor’s potential impacts, a systematic overview of studies examining the interactions when spontaneous humor is used during professional dialogue is currently absent. This review aims to delve into the interactive aspects of humor as a primary theme, linking humor with the coaching literature, distinguishing between the users of humor (whether it is the client, practitioner, or both), and the observed effects.

In conclusion, our study poses two specific inquiries:

1. What existing insights are available regarding humor use in interaction, and what are the effects of humor use in a one-to-one setting where professional dialogue is utilized for personal development?
2. What insights from adjacent fields can be applied to the coaching practice, and what factors should be considered when implementing humor in a coaching setting?

By addressing these questions, we aim to provide valuable insights for coaches, scholars, and practitioners alike, enhancing the understanding of the role of humor in coaching and its potential impact on professional development.

### 1.1 Coaching

In a rapidly evolving world where adaptability is key, coaching has become a popular medium to personally develop and keep up with society’s demands (Theeboom et al., 2017). It is firmly established within work contexts in Europe, the United Kingdom, Australia, and the United States (Joseph, 2016; Passmore et al., 2018). The field of “coaching psychology” has even emerged as a distinct domain in several universities (Grant and Atad, 2022). In this study, coaching is perceived as a dialogic intervention, in a one-to-one setting, aiming to help the client to address issues or problems, fostering the well-being and personal or professional development. Coaching, mentoring, counseling, and psychotherapy all fall under the umbrella of “professional helping relations” (Theeboom et al., 2014), however, differ in the type of clientele, subject matter, the use of diagnostics, goals of the interventions, and the stakeholders, such as family versus an executive team (Theeboom, 2021). Notably, the primary distinction between coaching and therapy lies in coaching’s focus on the client’s goal attainment and extends across domains such as executive, workplace, life, and personal development (Schermuly and Graßmann, 2019). To elaborate on the specifics of mentoring, a mentorship is a collaborative long-term relationship in which an experienced individual serves as a sounding board, offers support, and coaching, to help a less experienced person advance their career (Hill et al., 1989; Al Hilali et al., 2020). In Table 1, we present the summary of studied adjacent fields their main process focus, the nature of the relationship, and process.

**Table 1:** Three types of professional dialogue.

Psychotherapy/counseling	Mentoring	Coaching
Process focus		
Restore mental wounds, alleviate, and understand past	Career development, clarify organizational culture	Goals attainment, well-being, present, and future
Relationship nature		
Expert-patient	Tutor-junior or junior-tutor	Partner
Therapeutic, medical model	Practical advisory model	Educational developmental model
Long term	Long term	Short term
How		
Diagnosing, healing, catharsis, and investigate family of origin	Share experience, work related guidance, sounding board, and advice	Outcome, goals-setting, and action plans

Source: Adapted from Grant and Green (2018, p. 353).

All professional dialogues may incorporate humor; the key distinction lies in the primary focus and approach. In psychotherapy, humor is employed to facilitate healing, self-discovery, and insight. Humor can be integrated with clinical expertise and into therapeutic interventions, based on clinical assessments and treatment plans (Gelkopf, 2011). Humor in therapy can aid in emotional processing helping the clients express and make sense of complex feelings (Brooks et al., 2021). In coaching, humor is employed to create a positive and supportive environment for skill development. For example, Schinzilarz and Friedli (2013) used 26 humor interventions to achieve a lightened atmosphere and give insight into behavior. For instance by using props, images and caricatures, gibberish, word-salads, and smileys (Schinzilarz and Friedli, 2013). Coaches can utilize these action-oriented strategies, emphasizing accountability and results (outcome). Humor in coaching may serve as a motivational tool, promoting a positive mindset and resilience in the face of challenges: Dornaus (2016) states that in coaching counter conditioning with humor could be used, for example in the case of anger or stress reactions. Using humor as a diagnostic tool seems to make less sense in coaching; however, it seems sensible to consider the type of humor that suits the client and their professional environment (Dornaus, 2016). In all above mentioned professional dialogues, there is notice that (positive) humor serves as a relationship enhancer (Meyer, 2007; Love, 2013; Dornaus, 2016).

Provocative coaching, a specialized form of coaching with implications for humor, warrants attention. Derived from provocative therapy (Farrelly and Brandsma, 1981), several European psychologists adapted this therapy to coaching practice (Hollander and Wijnberg, 2006; Höfner, 2011). Infused with humor and paradoxical intention, this approach employs humor as a strategic tool to convey messages, increase client acceptance, and exhibit warmth and positive regard toward clients (Vendl, 2017).

### 1.2 Humor definition and humor styles

There are many definitions of humor. Humor is a biologically based, subjective, and social experience between humans. It is a social behavior and is seen as the ability of individuals, circumstances, or objects to elicit enjoyment or laughter between people (Richman, 1996). Although the definitions are dispersed there is a widespread understanding that humor involves the communication of more incongruous meanings that are amusing in one way or the other (Banas et al., 2011). Humor involves cognition, behavior, emotions, physiological processes, and social tasks. Five general theoretical approaches have been most influential in psychological humor research, namely psychoanalytic, superiority, arousal, incongruity, and reversal theory (Martin and Ford, 2018). It has been researched for its potentially protective and enhancing factors. It is protective in the sense that humor boosts positive moods and counteracts negative emotions. As an enhancing factor, humor plays a role in facilitating personal relationships, and this social relationship in turn plays a significant role in the use of humor in coping with life stress. Humor is not a unitary construct and may occur in many forms (Freud, 1971). Humor as adopted in this study is not telling jokes, with the client as the audience and the coach as the comic. Humor studied here arises in unplanned moments that occur spontaneously and is referred to as therapeutic humor. While humor interventions in professional dialogues are typically planned to aid clients, this study focuses on spontaneous, therapist-initiated humorous moments arising from empathic affective attunement, as distinct from structured interventions (Valentine and Gabbard, 2014). This humor is context-specific and sheds new light on an aspect of the client’s issue. It is an emergent, informal moment (de Haan et al., 2013) and is highly improvisational (Irving, 2019). Martin et al. (2003) developed a well-known model for the relation between types of humor and health, and a related measure. The Humor Styles Questionnaire (HSQ) differentiates four humor styles, two positive and two negatives, that might be beneficial or harmful to mental health. Self-enhancing and affiliative humor are called adaptive styles, while self-defeating and aggressive humor are considered maladaptive styles (Kuiper, 2016). Table 2 summarizes these styles.

**Table 2:** Four humor styles (HSQ).

	Adaptive humor styles	Maladaptive humor styles
Focus on self	Self-enhancing humor	Self-defeating humor
	“I’m often amused by absurdities of life, even when I am alone”	“I tend to put myself down if to make other laugh”
Focus on others	Affiliative humor	Aggressive
	“I like to laugh and joke a lot with my close friends”	“If someone makes a mistake, I will often tease them about it”

Source: Adapted from Kuiper (2016, p. 2).

### 1.3 Previous reviews on humor and coaching

Recent published reviews on humor have primarily focused on its effect on psychotherapy (Gelkopf, 2011; Brooks et al., 2021; Sarink and García-Montes, 2023), however also personal development (Gonot-Schoupinsky et al., 2020) and mental health (Schneider et al., 2018; Zhao et al., 2019). Two review studies explored humor only in a pediatric context and were excluded from our analysis (Haire and MacDonald, 2019; Sun et al., 2023). Table 3 provides an overview of these studies. Gelkopf (2011) centered on theoretical papers and book chapters from 1960 to 2000 emphasizing spontaneous humor use. He found substantial evidence supporting the therapist’s use of humor as an adjunct to conventional treatment, enhancing acceptance, empathy, and the therapeutic alliance. Spontaneous laughter can improve the patient’s trust in the therapist and process. Spontaneous humor of the client may help with diagnosis because jokes of clients can be a protective tool or an indication of the process of therapeutic change. According to Gelkopf (2011), the lack of humor research can be found in the historical focus (e.g., middle ages, the unholy trinity devil-folly-laughter were to be burned), emotional distance in psychiatric instances, conformity (humor is not considered mainstream, it has a “new age” side to it) and in the avoidance of risk (humor may negatively affect the client).

**Table 3:** Systematic review studies humor and coaching.

Author/Year/Journal	Studies	Context	Humor focus	Results
**Humor in therapy reviews**				
Sarink and García-Montes (2023)	10	Humor and outcomes in clinical population.	All humor program interventions but one: tailored laughter, movies, stand up, humorous outlook/skill, clowns, and humorous remarks of therapist.	Humorous interventions can have significant positive effects on symptoms of depression and anxiety.
*Frontiers in Psychiatry*				Research is based on different types of methods and population.
Brooks et al. (2021)	20	Use of banter in individual and group psychotherapy.	Banter related humor triggered by client and/or therapist.	One study only related banter to outcomes.
*Counseling and Psychotherapy Research*				Output theoretical categories: banter helps recognize feelings, helps start reciprocal process, allows creative energy between therapists, challenge preconceived and sacred notions, causes transformation, helps to make therapist less formidable, has destructive aspects.
Gonot-Schoupinsky et al. (2020)	564	Laughter and humor interventions, which optimize personal development in clinical and non-clinical population.	Mostly laughter and humor program intervention: learning, physical impact, therapeutic humor, humor in medical patients, humor in mental disease, clowns, films, and comedy training.	Characteristics of humor: universal, contagious, benefits, occurs alone, can be self-induced, trainable, association with playfulness/pleasure, has risks, harmful, can be drug-induced, influenced by context/location/individual and cultural differences and can be (in)voluntary and spontaneous/purposeful.
*European journal of integrative medicine*				Humor interventions can be tailored to therapeutic potential, and can be used for selfcare, integrative and complementary resources.
Zhao et al. (2019)	10	Effectiveness of laughter and humor interventions on depression, anxiety and sleep quality.	Laughter therapy, humor therapy or clown intervention on adults and/or medical workers	Synthesizing current evidence using laughter and humor interventions to reduce negative emotion and applicability between different adult populations and intervention methods. Humor and laughter interventions are a safe, convenient and interesting method, can promote interpersonal relationship between patients and medical workers and improve well-being in adults.
*Journal of advanced nursing*				
Schneider et al. (2018)	37	Correlations between HSQ and four areas of mental health.	Non-clinical population. Intervention focus not relevant.	Positive humor styles are overall positively correlated with mental health. Self-defeating humor is overall negatively associated with mental health. Gender and ethnic differences exist.
*Scandinavian Journal of Psychology*				
Gelkopf (2011)	23	Humor and laughter use in SMI individual and group therapy.	Humorous remarks therapist and client, laughter program, movies, stand up, humor interventions, clowns.	Can alleviate some of the daily distress by the SMI.
*Evidence-based complementary and Alternative Medicine*				Potential contributions: diagnostic, emotional, cognitive, somatic, space to play, release rigid defense, establish therapeutic alliance, maintains therapist mental health.
				Is easy-to-use and inexpensive.
**Coaching reviews**				
Wang et al. (2022)	20	Workplace coaching.	Learning, performance, psychological well-being.	Coaching facilitated positive outcome all focus area’s especially goal attainment, self-efficacy results commitment and job satisfaction. Using an integral approach as coach, with knowledge of psychology facilitates better outcomes.
*Journal of Work-Applied Management*		Investigating PIC (CBC-SFC)		
Richter et al. (2021)	24	Theoretical papers investigating which tools and techniques can be classified into model of Van Zyl et al.’s PPCM.	Positive psychology focus on tools and techniques coaches use.	PPC employ 18 types of PPC techniques and 117 coaching tools. Most tools should be employed in at least two phases of Van Zyl et al.’s model or continuous. Professional coaches and PPC differ in how tools/techniques should be classified.
*Frontiers in Psychiatry*				
Graßmann et al. (2020)	27	Studies with quantifiable measures of working alliance and coaching outcomes.	Affective, cognitive and result outcomes. Unintended negative effects.	Results steady over types of clients, coaches, expertise, number of sessions. Similar to psychotherapy and mentoring result supports importance of high-quality working alliance. Positive all outcomes, affective and cognitive strongest (r 0.32–r 0.64).
*Human Relations*				
de Haan (2019)	110	Qualitative research on workplace and executive coaching.	Understanding effectiveness of coaching: achievement, differences, impact on organizations practical implications.	Success is related to coach-coachee aspects and are:
				Trust, acceptance, and commitment to coaching and respect to contract from coachee.
*Consulting Psychology Journal*				Agreement on goals, shared psychological understanding and new insight.
Bozer and Jones (2018)	117	Workplace coaching determinants of effectiveness.	Determinants of coaching effectiveness and appropriate research methodologies.	Coaching is overall effective in organizations:
				Self-efficacy, coaching motivation, goal orientation, trust, interpersonal attraction, feedback, and supervisory support.
*European Journal of Work and Organizational. Psychology.*				Internal coaches do better than external.
				No difference between, face-to-face, e-coaching, or blended.
Theeboom et al. (2014)	18	Personal, group, peer and organizational coaching.	Effectiveness of coaching on individual level outcomes.	Positive effect on all focus topics from s 0.43 (coping) to s 0.74 (goal directed self-regulation).
*Journal of Positive Psychology*				Benefit from coaching for organizations: employees’ performance, skills, well-being, coping, work attitudes, and self-regulation.

SMI, Seriously mental ill; PIC, Psychologically informed coaching; WA, Working alliance; PPC, Positive psychology coaches; PPCM, Positive psychology coaching model.

Zhao et al. (2019) examined how humor interventions (laughter-, humor-, and clown programs) some in individual settings, and some in-group settings, contributed to the improvement of depression, anxiety, or sleep conditions. Positive effects were found in all three areas caused by improvement of the emotional state, e.g., creating a positive mood, optimistic thoughts, and diminishing rumination. Patients with mental disorders profit less from humor interventions. Interestingly the therapeutic efficacy of humor interventions is mainly derived from spontaneous laughter, triggered by positive emotions or external stimuli, and self-induced laughter (generated by oneself at will, after yoga or training).

Schneider et al. (2018) focused on humor styles concerning mental health, highlighting self-enhancing humor’s positive associations with optimism, self-esteem, and life satisfaction. Most research was conducted on healthy individuals, no spontaneous humor in a therapeutic setting or professional dialogue was researched. Self-defeating humor appears to be a correlate of emotional instability and negative effects. Aggressive humor is unrelated to mental health. Women tend to associate affiliative humor more with optimism, so there are sex differences in appreciating a style. Also, geographical differences occur, especially in utilizing aggressive and self-defeating humor for Asians vs. Western societies. Western society tends to see aggressive humor as more positive and self-enhancing humor as less negative in comparison with Asian societies. The authors conclude that using humor can be seen as a therapeutic skill that must be trained.

The review of Gonot-Schoupinsky et al. (2020) conducted an extensive review on humor and laughter, encompassing 564 studies involving 574,611 participants, addressing physical and psychological health, social and socioeconomic factors, environmental factors, and behavior. However, coaching articles were notably absent from their findings, despite coaching being a crucial personal development strategy within organizations (de Haan, 2016; Joseph, 2016; Solms et al., 2021). Only one article was found about the use of humor by the therapist and its effect on the interaction and process; playful humor enabled a 15-year-old to open up after 3 years of mutism (Nakanishi, 2017).

Brooks et al. (2021) published a review on a special form of humor: “banter,” which can be summed up as intended to provoke or make fun of someone in a playful but friendly way. Only one study was correlational, relating banter to the outcome of therapy. Other studies were organized by deductive content analysis and contain group and individual analysis of the spontaneous humor that arises. All studies but one (Fabian, 2011) were from before 2000. Humor’s effects are diverse, ranging from prompting clients to explore emotions, initiating participation, enabling creative expression, challenging fixed thought patterns, and fostering positive transformations. Additionally, humor humanizes the therapist, yet it can also lead to negative outcomes like hostility, suppression, and trivialization.

The most recent review of Sarink and García-Montes (2023) collected empirical data about humor interventions (humor with/without laughter, clowns, video, comedy training, and watching movies) with mentally ill adult clients (depression, anxiety). One article was about spontaneous humor use by the therapist (Panichelli et al., 2018) and described humor interventions; telling jokes and metaphors, giving provocative nicknames, and exaggerating beliefs.

The review studies on humor in Table 3 highlight challenges related to humor operationalization. Research design inconsistency and varying humor events are measured. Humor interventions encompass passively absorption of humor such as exposure to clowns or watching funny videos, while others aim to induce laughter through humor intervention programs or humorous perspectives. Two reviews incorporate papers about spontaneous humor use but consider group *and* individual therapy together when examining the effects (Gelkopf, 2011; Brooks et al., 2021). The other three reviews mainly incorporate humor interventions where clients have to absorb or learn humor techniques (Zhao et al., 2019; Gonot-Schoupinsky et al., 2020; Sarink and García-Montes, 2023). Sarink and García-Montes (2023) acknowledges the design inconsistency and advocates a holistic approach where humor’s various facets collaboratively contribute to enhancing clients’ overall well-being in the context of personal development.

Despite the rich information gathered on humor and professional dialogues, recent coaching reviews have overlooked the use of humor as a skill or quality (Theeboom et al., 2014; Bozer and Jones, 2018; de Haan, 2019; Graßmann et al., 2020; Wang et al., 2022). Despite the wealth of theoretical insights and established theories concerning humor’s potential impacts, no comprehensive overview of studies exploring interactions involving spontaneous humor in professional dialogue currently exists. The six reviews on coaching did not result in studies investigating humor use in the coach-coachee relationship. Coaches have a variety of tools at their disposal as a recent review on coaching pointed out: 24 publications on positive psychology coaching provided 117 different coaching tools (e.g., retelling a story as survivor, not victim, gratitude visit, focusing on the here and now, and appreciative interview). Remarkably, humor was omitted as a coaching tool underscoring a gap in the coaching literature (Richter et al., 2021).

In conclusion, this study addresses this gap by focusing on humor’s interactional aspects, bridging connections between humor and coaching literature. We aim to investigate humor use in the working dyad studying interactions where professional dialogue fosters personal development. Additionally, we seek insights from related fields to inform coaching practice and guide the integration of humor in coaching settings.

## 2 Methods

### 2.1 Search strategy and screening process

Our initial conceptualization involved an exploration of the literature on humor and coaches. However, the absence of robust research in this specific domain prompted a reconsideration of our research focus and questions. Consequently, we broadened our investigation to encompass the broader utilization of humor in professional dialogue. Subsequently, a discernible research gap in the coaching literature concerning humor became evident. Therefore, we undertook the task of reformulating our research questions by considering existing insights into the use of humor within professional dialogue. To extend the scope to the realm of professional dialogue, we introduced the question: What insights from related fields can inform coaching practices, and what considerations should be taken into account when incorporating humor in a coaching setting?

To enhance existing knowledge from adjacent fields on humor use to coaching we used a Systematic Literature Review (SLR) methodology, PRISMA, and conducted our search based on their guidelines (Moher et al., 2016). While our work follows the structure of a SLR, some aspects of this review lean more toward an Integrative Literature Review, as described by Rocco et al. (2023). For instance, our purpose was to use different literature streams to make meaning in the field of coaching, and in the presentation, we gave a coherent conceptual structuring of findings. Our research focus in the current study is articles including humor used by clients, practitioners, or both, in the context of a professional dialogue. As a starting point, we chose the publication date 2000 considering that in two decades we could have an overview of what has happened in this field. Starting our search in September 2021 our process continued until June 2023. Our review protocol unfolds as follows:

In phase 1, we initiated our search in the search engine Google Scholar using the main keywords “humor” and “coaching” spanning from 2000 until 2022. The initial search yielded a substantial number of hits (over 38.500). To refine our scope, we narrowed down the coaching types by specifying them, such as career coaching, executive coaching, workplace coaching, life coaching, and mental coaching. We further broadened the humor range by adding the keywords “humor intervention” and “playfulness,” the latter being closely related to humor and recognized as a special variant of play (Proyer, 2018). This resulted in a keyword string: [(“career coaching” OR “workplace coaching” OR “life coaching” OR “executive coaching” OR “mental coaching”) AND (“humor” OR “humor” OR “playfulness” OR “humor intervention”)]. Our focus was solely on peer-reviewed articles, with adult population mentioned and keywords being present in the abstract and/or title. This resulted in 346 results searching in Google Scholar, ProQuest Articles, and ProQuest Central. We added two articles through a reference search. After excluding articles related to horses, sports, teams, and couples and eliminating duplicate entries, only one empirical article remained about humor use in coaching. To ensure a comprehensive search, we consulted four established researchers in coaching, and their input confirmed the scarcity of empirical studies on humor and coaching.

**Figure 1:**
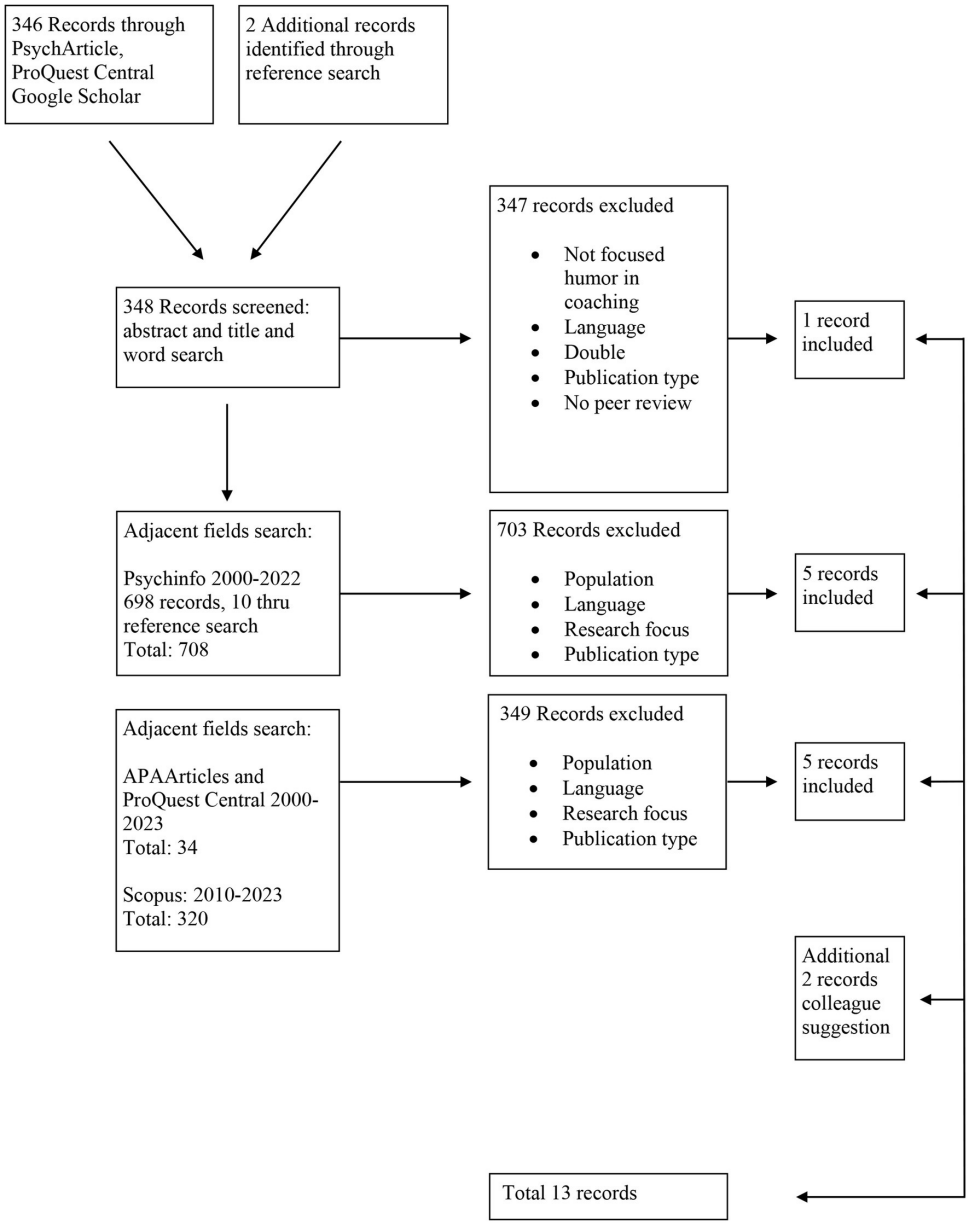
Search diagram.

Our next step (phase 2), starting in January 2022 was to search in adjacent literature, considering that psychotherapy, mentoring, and counseling (according to our reasoning explained earlier) shared a common ground with coaching as professional dialogues oriented to personal development. Starting in Psychinfo, a relevant database in Psychology, our search included keywords encompassing both the humor dimension (humor*, humor*, “humor use,” joy*, banter, and playful*) and the therapeutic dimension (psychotherapy*, therap*, and counsel*). This search was conducted for papers within the period from 2000 up to 2022, targeting peer-reviewed journal articles with adult populations. In October 2022, we found 698 papers and through reference search another 10. We underwent a content analysis of the titles and abstracts, excluding papers that were focused on a “humor intervention program” or listed in the aforementioned review studies on humor, aiming to make a distinct contribution to the field. Excluding articles related to couples and eliminating duplicate entries, five qualitative and/or quantitative studies were identified. Acknowledging the close relationship between mentoring and coaching (Passmore et al., 2012; Drake, 2021), we conducted two other searches. In January 2023, a search in Scopus was conducted with the following thread [(“humor*” OR “humor*” OR “playful*” OR “banter*” OR “using humor”) AND (“psychotherap*” OR “counsel*” OR “mentor*”)]. Resulting in 320 hits. After reading the articles, title, and abstract we excluded: telephone counseling, virtual reality counseling, therapy conducted on people with dementia, or research aimed at group settings. We always excluded articles that were about humor training or programs but hand-searched whether there was spontaneous humor in the interaction of the dyad. The search gave us five new papers. In July 2023, we searched Pro Quest Central and APA Psych Articles searching with the thread [(“humor*” OR “humor*” OR “playful*” OR “banter*” OR “humor intervention”) AND (“psychotherap*” OR “counsel*” OR “mentor*” OR therap*)] in the title. We excluded articles about children and the word “humoral” because that is a medical condition. This gave 34 hits and did not result in a new record. Throughout this process, we remained receptive to input from colleagues, resulting in the addition of two more articles to our final selection (Figure 1).

### 2.2 Eligibility criteria

#### 2.2.1 Language

Only papers written in English and German were subjected to scrutiny.

#### 2.2.2 Research focus

Only papers where the humor use of a client and/or therapist was the focus of the research were included. Papers exclusively centered on humor-enhancing programs were excluded. Additionally, papers solely addressing laughing as a behavioral expression (e.g., measuring smiles) not directly related to humor, were excluded.

#### 2.2.3 Population

Both clinical and non-clinical populations were considered, while populations with dementia, and individuals under the age of 18 were excluded.

#### 2.2.4 Publication type

Only peer-reviewed articles were included, while conferences and book chapters, dissertations were excluded. Only primary studies were included, so systematic reviews and meta-analyses were excluded.

#### 2.2.5 Setting

Only face-to-face settings were enclosed, excluding telephone counseling and/or email or other chat therapy. Sports, team, and couples settings were excluded.

The reviewed papers were published between 2013 and 2023 in 11 journals spanning four continents. The number of participants in the selected studies varies from 1 to 110 participants (Table 4).

**Table 4:** Characteristics of the studies reviewed.

Study number	Authors	Title and Journal	Country	Sample
**Quantitative studies**				
1	Panichelli et al. (2018)	Humor associated with positive outcomes in individual psychotherapy. *American Journal of Psychotherapy*	Belgium	One therapist
				110 clients
2	Graßmann et al. (2020)	Welche Strategien nutzen Coaches bei herausforderung Klienten? Eine explorative Analyze von Herausforderungen, Strategien und der Rolle van Supervision. *Coaching Theorie & Praxis*	Germany	99 coaches
3	Love et al. (2020)	The influence of humor on workplace mentoring and employee attitudes. *Social Behavior and Personality*	United States	54 mentors
				54 mentees
4	Kneisel et al. (2022)	Humor and laughter in counseling: a content analysis of 39 videorecorded counseling sessions. *Journal of Creativity in Mental Health*	New Zealand	37 counselors
				37 clients
5	Brooks et al. (2023)	Banter in psychotherapy: relationship to treatment type, therapeutic alliance and therapy outcome. *Journal of clinical Psychology*	Austria	18 therapists
				68 clients
6	Morrison and Smith (2013)	Working alliance development in occupational therapy: A cross-case analysis. *Australian Occupational Therapy Journal*	Canada	Two therapists
				Four clients
Qualitative studies				
7	Scott et al. (2015)	The healing power of laughter: the applicability of humor as a psychotherapy technique with depressed and anxious older adults*. Social Work in Mental Health*	United States	One therapist
				One client
8	De Lange and Chigeza (2015)	Fortigenic qualities of psychotherapist in practice. *Journal of Psychology Africa*	South Africa	Seven therapists
9	Dionigi and Canestrari (2018)	The use of humor by therapist and clients in cognitive therapy. *European Journal of Humor Research*	Italy	Eight therapists
				Eight clients
10	Gibson and Tantam (2018)	The best medicine? Psychotherapists’ experience of the impact of humor on the process of psychotherapy. *Existential Analysis*	United Kingdom	Six therapists
11	Schapiro-Halberstam et al. (2020)	Young women treating men with borderline personality disorder: a challenge for psychotherapy integration. *Journal of Contemporary Psychotherapy*	United States	One therapist
				One client
12	Hussong and Micucci (2020)	The use of humor in psychotherapy: Views of practicing psychotherapist. *Journal of Creativity in Mental Health*	United States	10 therapists
13	Briggs and Owen (2022)	Funny, right? How do trainee and qualified therapist experience laughter in their practice with clients? *Counseling and Psychotherapy Research*	United Kingdom	Six therapists

### 2.3 Risk of bias

To minimize the risk of bias, we adhere to the review steps outlined by PRISMA (Moher et al., 2016). Although our study has some characteristics of an Integrative Literature Review (ILR) like a more reflective analysis and some coherent conceptual structuring of the results, this article mostly follows the structured and detailed method of a SLR that can be replicated (Rocco et al., 2023). This evidence-informed methodology minimizes the risk of results being inadequate or incomplete. By using a step-by-step selection process, the quality of the underpinning of our results is assessed and it can be easily replicated for other researchers.

#### 2.3.1 Publication bias

Our study is shaped by the search engines we used. Looking into search engines that are mostly psychologically informed, Psychinfo, Scopus, and APA articles, we lack articles found in more management search engines, which may induce a publication bias.

### 2.4 Studies characteristics

The studies reviewed were conducted using quantitative, qualitative, and mixed-method approaches. Diverse outcome measures were employed, encompassing a wide range of potential outcomes. To assess humor use in professional dialogue, most studies used semi-structured interviews (studies 6, 8, 10, 12, 13) and content analysis of predominantly video sessions (studies 4, 5, 9, 11, and 13). One study (3) focused on observing the interaction of both the client and the practitioner. In addition, one study (6) also gathered information from clients. There were two (7 and 8) descriptive case studies where authors described their session with a client while using humor (see Table 4). Our study shows similarity in using different methodologies (e.g., case study, mixed method, observational methods, and RCT) with the review studies on humor like Brooks et al. (2021), Gonot-Schoupinsky et al. (2020), and Gelkopf (2011), providing a rich detail by using a balanced mixture of methodologies.

Participants encompassed adult clients (283) and/or practitioners (250). Eight of the 13 studies combined clients and practitioners. These studies examined the effects of humor used by both, clients and practitioners, emphasizing the interactional impact. The practitioners represented various roles, including psychotherapists (studies 2, 5, 6, 7, 11), therapists with diverse orientations (studies 10, 13), cognitive behavioral therapists (study 4), mentors (studies 3, 9), coaches (study 8), counselors (study 12), and occupational therapists (study 1). The studies were predominantly conducted in Western societies, spanning countries like Canada, the United States (5), South Africa, Italy, the United Kingdom (2), Belgium, New Zealand, and Germany. Due to variations in research design across the selected studies, the distinct outcomes of each study are presented in Table 5: Studies 1 and 2 were quantitative studies, studies 3–5 used a methodological approach of mixed methods, and 6–13 were qualitative approaches.

**Table 5:** Overview of studies on humor use in practitioner-client relation.

*N*	Ref. date	Method of data collection	Who uses humor + Why	Outcomes	Profession
	**Methodological approach: quantitative**				
1	Panichelli et al. (2018)	Questionnaires HDRS HTQ CGI	Practitioner: tool to lower anxiety; maintain therapeutic alliance; increase pleasure in therapy.	Practitioner using spontaneous humor had effect on outcome, causality not known. Only interventions targeted on clinical outcome and problem must be used. Decrease of humor in patients with severe problems. Clients with lower CGI less humor in session. Client humor score neg related CGI.	Psychotherapist
			Client: avoidance; make situation less painful; escaping confrontation.		
2	Graßmann et al. (2021)	Online questionnaire	Practitioner: tool to deal with difficult client.	Humor used as strategy for difficult clients.	Coach
	**Methodological approach: mixed methods**				
3	Love et al. (2020)	Questionnaires GJSS AOCS RSGS PMHS Mentoring satisfaction Humor frequency	Practitioner: tool to foster alliance	Antecedent to successful mentoring. Frequent use greater satisfaction. Work related outcomes; relation, job satisfaction, commitment turnover.	Mentor
4	Kneisel et al. (2022)	Content analysis session	Practitioner: tool to connect; break down power balance; gently deliver feedback; challenge stereotypes; confront believes; overcome cultural differences; and lighten the mood.	Laughter was always present. Client laughed more than practitioner. Gender did not play a role in humor initiation, ethnicity matters.	Counselor
			Client: mediate anxiety; get reassured by practitioner.		
5	Brooks et al. (2023)	Content analysis and questionnaires BDI IIP INTREX p/n SCL-90-R F-SOZU	Practitioner: facilitation and confrontation	Humor present 4.5 times per session. Most humor in CBT session. Humor predicted therapy outcome; correlation between humor and “bond.” Therapist needs to pay attention to the client response. Negative banter/humor was risky and hurtful.	Psychotherapist
			Client: defense mechanism		
6	Morrison and Smith (2013)	Case study Questionnaires OSA WAI	Practitioner: enhance comfort, diffuse tension, ease path in difficult process for client; establish working alliance.	Humor present in all sessions. Humor use developed WA had therefor effect on outcome through client’s ability to do everyday tasks with greater ease.	Occupational therapist
			Clients: facilitates relation, bonding; use as emotion regulator.		
	**Methodological approach: qualitative**				
7	Scott et al. (2015)	Case study	Practitioner: using humorous statements for cognitive re-shift, lighten atmosphere.	Older patients loved lightness and perspective. Humor helped seeing life in benign way.	Psychotherapist
				Humor helped feeling less afraid being vulnerable in therapy.	
8	de Lange and Chigeza (2015)	Exploratory conversations/interviews	Practitioner used as a coping skill to Remain calm. Stay focused. Relieve exhaustion and burnout.	Humor was helpful tool against compassion fatigue.	Psychotherapist
9	Dionigi and Canestrari (2018)	Session analysis	Practitioner: make situation les charged; bring message softly; facilitate trust; humanizing, relax the client; ease difficult communication.	Humor initiated by both was an important resource in DBT. When practitioner uses humor, client always laughed, the other way around not.	CBT therapist
			Client: reducing emotional stress; expression of frustration and coping mechanism; as emotional outlet.	Practitioner must learn how and when to use it.	
10	Gibson and Tantam (2018)	Interpretative phenomenological analysis (IPA)	Practitioner: tool to stablish work relationship, cognitive and behavioral shifts; catalyze, lead to further exploration; targeting problem; working alliance.	Thoughtful use of humor in therapy promoted existential maturity and humorous attitude, allowing creative acceptance of limitations and paradoxes. Mistiming or defensive use of humor may impede the progress of the psychotherapeutic process.	Psychotherapist
			Client: feeling better in the contact.		
11	Schapiro-Halberstam et al. (2020)	Case study with progress coding	Practitioner: tool to deal with difficult client; confront; break through manipulation.	Irreverent humor significantly declined out of control sexual behaviors. Helped to overcome the stereotype female role expectations. Risk: shame-sensitive clients feel rejected and judged.	Psychotherapist
12	Hussong and Micucci (2020)	Interview	Practitioner: tool for assessing capacities; reducing defensiveness enhance alliance; build up self-awareness; modeling adaptive behavior; encouraging flexibility; diagnostic.	Use is beneficial unanimous regardless practitioner different theoretical backgrounds. Advantage when client uses: gave insight in capacities and personality dynamics.	Psychotherapist
			Client: modality to cover up painful feelings.	Risks when practitioner uses: offending, covering up, to forced, boundaries fluid, gender/cultural bias, and countertransference.	
13	Briggs and Owen (2022)	IPA—semi structured interviews	Practitioner: Foster the working alliance by following client’s lead, let client feel safe. Tool for the process, catharsis, addressing power, as a sign of progress.	Humor was highly beneficial for relationship, facilitates positive outcome (creativity and catharsis). Should be topic of counseling courses. Gallows humor had a function. Client’s humor use gave information about mindset. Defensive humor was detrimental.	Psychotherapist
			Client: sign of progress, sign of creativity & playfulness, deal with adversity and trauma (gallows humor).		

HDRS, Hamilton depression rating scale; HTQ, Humor in therapy questionnaire client and therapist version; CGI, Clinical global impression scales; HDRS, Hamilton depression Scale; HTC, Humor in therapy questionnaire; OSA, Occupational self-assessment; WAI, Working alliance inventory; BDI, Beck depression inventory; SCL-90-R, Symptom checklist-90-rvisited; IIP, Inventory of interpersonal problems; F-SOZU, Social support questionnaire; INTREX p/n, Introject questionnaire positive/negative; S-WT, Shapiro–Wilk normality test; WSR, Wilcoxon Signed Rank test; GJSS, Global job satisfaction scale; AOCS, Affective organizational commitment scale; RSGS, Revisited stress in general scale; PMHS, Positive mentor humor scale; and KBI, Klagenfurt inventory instrument.

The focus of the studies could be classified into two main areas: humor use by the therapist/coach/mentor (4 of 13) or both (9 of 13). This division resulted in research investigating humor’s presence in therapy sessions, whether the humor was initiated by the practitioner or the client, and the effect of humor when used by the practitioner or the client. Additionally, when we could not analyze who made the humorous remark, we concentrated on studying humor as “being present” in the session and its effect on the interaction. Subsequently, we analyzed the reported effects of humor on theoretically based categories. These results are shown in Table 6. Additionally, we present results in terms of humor styles of Martin et al. (2003).

**Table 6:** List of outcome categories in professional dialogues.

Category name	Frequency	References
Well-being of the client	8	Morrison and Smith (2013); Scott et al. (2015); Dionigi and Canestrari (2018); Panichelli et al. (2018); Hussong and Micucci (2020); Graßmann et al. (2021); Briggs and Owen (2022); Kneisel et al. (2022)
Fostering working alliance	8	Morrison and Smith (2013); Gibson and Tantam (2018); Hussong and Micucci (2020); Love et al. (2020); Graßmann et al. (2021); Briggs and Owen (2022); Kneisel et al. (2022); Brooks et al. (2023)
Effects on energy, creativity, and depth	7	Morrison and Smith (2013); Dionigi and Canestrari (2018); Gibson and Tantam (2018); Hussong and Micucci (2020); Schapiro-Halberstam et al. (2020); Briggs and Owen (2022); Kneisel et al. (2022)
Cognitive and behavioral shifts	7	Scott et al. (2015); Gibson and Tantam (2018); Panichelli et al. (2018); Hussong and Micucci (2020); Briggs and Owen (2022); Kneisel et al. (2022); Brooks et al. (2023)
Effects on the practitioner	4	De Lange and Chigeza (2015); Schapiro-Halberstam et al. (2020); Graßmann et al. (2021); Kneisel et al. (2022)

### 2.5 Data extraction

The complete texts of the 13 studies included in this review were utilized to construct the database. To offer a systematic overview, we extracted essential details necessary for clear identification of the papers, along with information pertinent to addressing the two research questions. Table 4 represents more bibliographical information, country of publication, and how many participants the studies contained. Table 5 represents the following information from the included studies: contextual details such as methodological approach, data collection methods, operationalization humor use in the practitioner-client relation, potential outcomes explored, and corresponding findings. The challenges we faced were that the participants and the method varied to a large extent. Utilizing data from studies with clinical and non-clinical populations, incorporating research from mentoring, psychotherapy, and counseling all have slightly different approaches regarding the focus of the process, the nature of the relationship, and how the professional proceeds (Table 1).

## 3 Results

### 3.1 Quantitative studies

Our results of outcomes are presented in Table 5. Quantitative studies showed that humor was related to positive outcomes (studies 1, 3, 4, 5, and 6). Panichelli et al. (2018) found a negative correlation between par Clinical Global Impressions (CGI) scores and the presence of humor in therapy, indicating that the therapist rated the presence of humor higher when there was more improvement in therapy. Additionally, when patients had more severe problems, there was less humor, and clients who perceived the therapist as less funny had lower hope and pleasure scores although this did not affect the outcome. While this was not a randomized trial, this exploratory study demonstrated an initial, positive finding for the use of humor in a clinical setting. In the study of Brooks et al. (2023), the INTREX Positive Introject Outcome was statistically predicted by humor, suggesting using humor led to positive internalization of others’ attitudes toward the client. The correlational in this study (e.g., study 5) on humor and working alliance (WA), showed humor did not have a significant impact on the overall quality of the WA, but the subscale bond (emotional connection and trust) had a significant correlation between WAI-SR (*r* = 0.26; *p* = 0.035), meaning that banter contributed to the connection and trust. Further empirical data on actual instances of humor (banter), proved that laughter occurred in most sessions with 4.54 actual instances per session (Brooks et al., 2023). In a study related to mentor satisfaction (Study 3), researchers analyzed the relationship between humor, mentoring satisfaction, affective organizational commitment, job satisfaction, and turnover intentions. A positive humor style was associated with increased mentoring satisfaction (*p* < 0.001) and humor frequency was positively related to mentor satisfaction (*p* < 0.001). A higher level of mentoring satisfaction was associated with increased organizational commitment, job satisfaction, decreased turnover, and a significantly positive relationship with affective commitment (Love et al., 2020). Study 4 focused on who laughed more, the practitioner or client, and what the target was of laughter (Kneisel et al., 2022). They found that clients laughed more than practitioners and practitioners’ laughter was more shared laughter than laughing alone. The laughter was greater at the beginning of the session. There was more laughter in different ethnic dyads. Practitioners laughed most at human nature (36%) and targeted the client (51%). Clients targeted themselves and laughed most at themselves (36 and 58%). In the study of Morrison and Smith (2013), the conclusion was that humor helped to foster an alliance and this had an effect on outcome: clients showed significant improvements in everyday tasks, which in turn boosted their self-confidence.

### 3.2 Qualitative results

Alongside the quantitative outcome, this section is organized by theoretically based categories. Since our second research question was about gaining insights from related fields that can inform the coaching literature, qualitative literature is helpful for development of theory. Derived from on careful reading of all 13 studies, our review involves the establishment of categories through an initial inductive content analysis of the results. Subsequently, we conducted a deductive content analysis checking whether these categories were familiar and also appeared in our aforementioned review studies on humor, which indeed affirmed. The content analysis presented in this section is central to obtaining a more profound qualitative comprehension of when and how humor manifested itself in a professional dialogue. The categories might overlap because humor can affect one category through the other. We found that humor has been found to have effects in five areas, which are categorized by the overall topics of humor use by practitioners and/or clients. These areas are (1) enhancement of client well-being, (2) augmentation of the working alliance, (3) amplification of energy, creativity, and depth, (4) catalysis for cognitive and behavioral shifts, and (5) impact on the practitioner. Table 6 shows the number of studies that mentioned the effects of humor on each category.

#### 3.2.1 Well-being

Both the client and practitioner used humor to enhance the well-being of the client. We identified eight studies in which humor was used as a coping mechanism to alleviate stress for the client. Practitioners strategically employed humor to create an atmosphere of comfort and lightheartedness (Morrison and Smith, 2013; Scott et al., 2015; Dionigi and Canestrari, 2018; Panichelli et al., 2018; Hussong and Micucci, 2020; Graßmann et al., 2021; Briggs and Owen, 2022; Kneisel et al., 2022). Clients often turned to humor to deflate the gravity of their issue and by that reduce stress. For instance, one client made fun of himself, called his visit to another practitioner a “*shock wave effect so to speak*” and started to laugh. Dissatisfied with the diagnosis he got from the former practitioner, the client’s remark aimed to lighten the burden (Dionigi and Canestrari, 2018, p. 55). This perspective also aligns with a reduction in client defensiveness. Hussong and Micucci (2020) found that all interviewed practitioners in the study noted that humor reduced clients’ defensiveness, allowing them to feel safe and therefore daring to speak about challenging topics. Study 4 showcased various instances where clients used humor to de-escalate their distress, as observed in analyzed videos (Kneisel et al., 2022).

#### 3.2.2 Working alliance

Eight studies (2, 3, 4, 5, 6, 10, 12, and 13) specified that there is a link between humor use in the session and the WA, also mentioned as a bond or therapeutic relation (Brooks et al., 2023). Humor is seen as a unique alliance builder between practitioner and client (Morrison and Smith, 2013; Gibson and Tantam, 2018; Hussong and Micucci, 2020; Love et al., 2020; Graßmann et al., 2021; Briggs and Owen, 2022; Kneisel et al., 2022; Brooks et al., 2023). Additionally, practitioners employ humor strategically, particularly when working with challenging clients, to enhance this bond. In a study involving 99 coaches, 48.5% of respondents reported using humor as a strategy with difficult clients, defined as those lacking awareness of their issues or diagnosed with mental disorders (Graßmann et al., 2021). This aligns with a case study where a young female practitioner learned to use irreverent humor to strengthen the client relationship, facilitating greater client vulnerability to process through his therapy topics (Schapiro-Halberstam et al., 2020). Kneisel et al. (2022) found after analyzing 39 recorded counseling sessions that humor was used to foster a positive therapeutic relationship between client and counselor, although it did not specify whether client or practitioner humor was involved. In study 6, a practitioner mentioned “It’s a fun relationship because he’s got good humor…” indicating that the client using humor facilitated the alliance (Morrison and Smith, 2013, p. 330). However, the use of humor also showed risks for the WA by promoting effects such as seducing or offending the client (Morrison and Smith, 2013; Gibson and Tantam, 2018; Panichelli et al., 2018; Hussong and Micucci, 2020). The alliance could be compromised by forced humor or by using humor with clients struggling to grasp double meanings (Hussong and Micucci, 2020).

#### 3.2.3 Energy, creativity, and depth

Another valuable lens through which to examine the process is to assess the impact of humor on the energy and depth of therapy sessions. Seven studies (4, 6, 9, 10, 11, 12, and 13) highlighted how humor influenced both positive and negative dimensions of session dynamics including energy levels and conversational depth. On the positive side, humor made clients ready for the demanding aspect of therapy and provided practitioners with an alternative route to proceed in response to difficulties in the interaction (Dionigi and Canestrari, 2018). In the study, six practitioners used humor to give an alternative way to proceed if the previous interaction was a difficult one. This is in line with conclusion of Morrison and Smith (2013, p. 332): “to use humor as a means to facilitate the arduous aspects of therapy.” In study 13, all six therapists mentioned that humor made it possible to meet each other on a deeply connected level, with one therapist stating, “Having that laugh together just feels like you are meeting at that point in that relational depth because there is something so palpably shared” (Briggs and Owen, 2022, p. 6). The introduction of humor was associated with more creativity and a variety of responses as mentioned by Kneisel et al. (2022). This observation aligns with the conclusions drawn by Hussong and Micucci (2020), who found that humor fostered an atmosphere of flexibility and playfulness during sessions. Clients can use humor as a catharsis enabler. One example illustrates a client’s laughter escalated to tears, an occurrence that led the practitioner to remark: “previously unacknowledged wounds came to surface” (Briggs and Owen, 2022, p. 6).

However, there are instances of negative repercussions. When the client was using humor and the practitioner went along, it sometimes veiled the exploration of profound emotions by concealing them with humorous remarks (Gibson and Tantam, 2018). An interviewed therapist points out this concealing effect: “There is no observing ego that informs either the patient or therapist about the hostile destructive effect of their use of humor. This feels like business-as-usual” (Gibson and Tantam, 2018, p. 72). Moreover, there existed a potential risk of adverse outcomes when practitioners used humor that was misunderstood, used to belittle, mimic, or laugh at the client (Dionigi and Canestrari, 2018).

#### 3.2.4 Creating shifts

The influence of humor on cognitive and behavioral shifts in clients was noted in seven studies (1, 4, 5, 7, 10, 12, and 13). Among therapeutic modalities, Cognitive Behavior Therapy (CBT) sessions emerged as particularly conducive to humor integrations contrasted with psychodynamic or psychoanalytic therapy. This approach within CBT facilitates the opening of clients to novel perspectives (Brooks et al., 2023). Kneisel et al. (2022) presented an additional cognitive benefit; after humor use, the client’s emotional state and evaluation of a situation changed. For instance, the study of Briggs and Owen (2022) showcased a client first reluctant to do the exercise “scream together.” However, after the practitioner used humor, the client displayed a greater willingness to openly discuss the feelings of being controlled, reflecting a newfound insight. Such findings resonate with study, study of Hussong and Micucci (2020) where seven out of 10 practitioners reported that humor, with the right timing, could lead to an exploration of clients’ self-consciousness or unease about certain topics. Furthermore, client-initiated humor emerged as an indicator of transformative change. A practitioner reflected: “I think any positive shift you witness in a client, obviously is brilliant as therapist (…) to see a client maybe go from being distressed and working their way through it, up to begin to see humor in something. It’s a really nice journey, actually to be a part of it” (Briggs and Owen, 2022, p. 7).

However, a potential drawback was identified in study 12, suggesting that when practitioners laughed along with clients using humor defensively, it could reinforce the avoidance. This resulted in clients employing humor as a coping strategy, not as an insight strategy. Furthermore, in cases where clients exhibit cognitive impairments or signs of disorders, caution was advised as it did not yield greater insight in such instances (Hussong and Micucci, 2020).

#### 3.2.5 Effects of humor use on the practitioner

The incorporation of humor extended the practitioner’s toolkit for managing challenging clients (Graßmann et al., 2021). Humor helped practitioners in navigating demanding scenarios like compassion fatigue as it facilitated composure and acted as a preventive measure against burnout (De Lange and Chigeza, 2015). This is in line with the case study of Schapiro-Halberstam et al. (2020), where humor helped the practitioner to set boundaries and therefore found herself in control of the session again, preventing herself from emotional exhaustion and female stereotyping behavior. Furthermore, the exploration of humor within clients’ familial contexts offered practitioners a window into understanding family dynamics and the coping mechanisms employed by the client (Kneisel et al., 2022).

### 3.3 Humor styles

In the subsequent section, we present the use of the *humor type* by clients or practitioners. Our way of analyzing the humor type is by using Martin et al. (2003), HSQ and description (Table 2). Through our analysis of our selected articles, we identified instances where humor styles as categorized by Martin were mentioned.

Practitioners predominantly favored adaptive humor styles. Affiliative humor was referenced in nine of our 13 studies (1, 3, 4, 5, 6, 10, 12, and 13). Affiliative humor gave a positive atmosphere which influenced WA (Morrison and Smith, 2013; Gibson and Tantam, 2018; Panichelli et al., 2018; Hussong and Micucci, 2020; Briggs and Owen, 2022). Self-enhancing humor was mentioned in seven of our studies (3, 4, 8, 9, 10, and 12). Maladaptive humor was less used. In two studies, practitioners used aggressive humor: One female therapist used it to establish a collaborative relationship where she had to be empathetic yet confrontative (study 11). In another study, gallows humor was used so the therapist could deal with severe trauma of the client (study 13). Self-defeating humor used by the practitioner was detected in Study 4 to break down the power balance they commonly have over their clients, while Study 12 highlighted that self-defeating humor made clients feel safe because the therapist was human.

Clients it appears leaned more toward self-defeating humor to cover up painful emotions (study 4, 9, 12, and 13). We found four studies with clients using aggressive humor: In study 5, client used it as a defense to cover up painful feelings, while in study 9, a client expressed frustration and sought goodwill from the therapist. Study 13 noted the use of aggressive humor as a coping strategy for severe trauma, and study 10 reported a client using aggressive humor to seduce the therapist. Aggressive humor also served as a form of catharsis (Briggs and Owen, 2022; Brooks et al., 2023). Only two of our studies (6 and 12) mentioned clients using affiliative humor, with clients employing this style to facilitate the relationship. In one instance (study 1), self-enhancing humor used by the client was linked to stress reduction.

## 4 Discussion

The beneficial implications of humor in professional dialogues have been emphasized in the literature since the 1970s, alongside warnings about its downsides. Our systematic review of 13 studies addressed a specific use of humor: the spontaneous humor that is almost always present in professional dialogues (Love et al., 2020; Brooks et al., 2023) but has not yet been studied systematically (Liebman, 2022). Our findings are consistent with recent reviews on humor, which suggest that the exploration of potential outcomes related to humor use in professional dialogue has gained significant attention in recent years. However, a notable gap exists in the coaching literature on this topic. Notably, most studies included in this review have a qualitative component that helps us to unpack what happens in a professional dialogue concerning humor use. This highlights the importance of investigating this positive emotion within the context of coaching and coaching education. Our included studies are not included in previous reviews except one (Panichelli et al., 2018).

### 4.1 Prevailing data

Much like the observations drawn from prior reviews, the included studies supported the relationship between humor use and outcomes in the professional dialogue context. These effects encompassed benefits for clients with mental disease diminishing their illness (depression), giving clients emotional alleviation and self-confidence. But also, organizational outcomes like job satisfaction and commitment, and turnover intentions are reported. Yet, the intricacies of how these outcomes are influenced by humor, particularly if humor serves as a mediating factor that enhances satisfaction with practitioners and subsequently contributes to favorable outcomes, remain ripe for exploration.

Another recognized advantage is the establishment and firming of the working alliance (WA). A novel insight of this review is that of “timing” and the specific aspects where humor proves effective. Contrary to prior reviews, our findings indicate that it may not be imperative to delay the use of humor until the establishment of the therapeutic bond. This notion is echoed by Kneisel et al. (2022), who observed heightened instances of laughter in the initial stages. This may be attributed to both parties’ shared endeavor to establish a secure connection. This novel perspective challenges the prevailing notion that humor should only be deployed once the therapeutic relationship is solidified. Our in-depth analysis of relevant studies indicated this result (Scott et al., 2015; Dionigi and Canestrari, 2018; Love et al., 2020). Furthermore, humor helps to foster a “bond” as empirical evidence is displayed (Love et al., 2020; Brooks et al., 2023). The three elements delineating the WA, as pointed out by Theeboom (2021) include shared responsibility, collaborative control of the therapeutic process, and mutual affinity and trust between coach and client. Our research underscores that humor likely exerts a notable influence on the third element, namely “trust and acceptance.” This warrants further exploration as it potentially holds significant implications.

Humor fosters cognitive and behavioral shifts as has been acknowledged in review studies (Gelkopf, 2011; Gonot-Schoupinsky et al., 2020; Brooks et al., 2021). In our review, seven studies mentioned shifts related to the influence of humor in professional dialogue. These shifts were mainly improvement of cognitive abilities, more creativity (Hussong and Micucci, 2020; Kneisel et al., 2022), and a variety of responses (Kneisel et al., 2022) which challenge and beliefs’ changes (Gibson and Tantam, 2018; Kneisel et al., 2022). Further research endeavors should delve into the mechanisms underpinning these outcomes. Sarink and García-Montes (2023) propose that humorous interventions contain surprise, momentarily disorienting clients. Clients are provoked to reassess established thought patterns and must go to another framework to resolve their confusion. We supplement this by proposing that a cheerful atmosphere could bolster creativity by fostering uninhibited thinking, thereby extending the resources available for different solutions. The effect of a cheerful temperament on creativity is reported by other authors (e.g., Lau et al., 2022) and applicable in coaching. Wheeler (2020) elucidates in her research on coaching and playfulness that a positive playful atmosphere encourages a more relaxed mindset, fostering clients’ innovative thinking and willingness to explore new ideas. Because of this willingness, clients are invited to question their habitual approach and dare to experiment with different behavior.

Another unique finding from our review centers on the consideration of aggressive humor. Three of our studies (Schapiro-Halberstam et al., 2020; Briggs and Owen, 2022; Brooks et al., 2023) underscore the role of more confrontational humor. Specifically, these studies highlight how such humor functions for clients, providing an avenue to fully express themselves. Additionally, for practitioners, aggressive humor serves as a valuable tool to manage interactions with challenging clients. This is in line with a recent study by Yonatan-Leus et al. (2018) where clients had positive therapy outcomes when the therapist used an aggressive form of humor. Building on findings of Brooks et al. (2023), getting a deep qualitative understanding of negative humor an explorative study could elaborate on where the line is between harmful and useful humor for the client and the process.

### 4.2 Directions for future coaching research

How do our findings resonate with the field of coaching? This sub-section will provide directions based on our second research question “What are the transferrable results from adjacent fields to the coaching practice and what are the points to consider?” Our first recommendation is advocating for qualitative research designs, uncovering the previously unknown outcomes of humor use, and preparing the ground in less-explored areas of research, as we will elaborate on below. Editorial of Hartman (1990) highlights the need for multiple approaches to understanding humor in coaching. While quantitative data can be used to measure the presence and frequency of laughter after a humorous interaction (Dionigi and Canestrari, 2018); it cannot provide insights into the intentions of the coach or client. To fully understand the complex phenomenon of humor, we must turn to observational methods, mixed methods, or phenomenological analysis (Del Giacco et al., 2020). These approaches can help us better understand the process through which humor intervenes in coaching and its impact on the client.

Most of the studies indicate that humor contributes to building an alliance, but there is a lack of understanding of the mechanisms by which humor contributes to the WA in coaching. While direct influence on session-to-session improvement is sometimes debated (de Haan et al., 2020), WA remains a crucial predictor of successful coaching (Theeboom, 2021). Positive working relationships positively impact coaching satisfaction, effectiveness perception, self-efficacy, knowledge acquisition, and self-reflection (Graßmann et al., 2020). Longitudinal studies also emphasize the significance of the WA (Molyn et al., 2022). Our findings suggest that a subcategory of WA, “bond” plays a crucial role (Love et al., 2020; Brooks et al., 2023). Review studies on coaching highlight trust as a variable of relevance for effective coaching (Bozer and Jones, 2018; de Haan, 2019). Although trust and bond are not the same constructs, they are related, and positive emotions play an important role in strengthening bonds and building trust (Sels et al., 2021). Authentic humor is found to strengthen the WA (Valentine and Gabbard, 2014) while forced humor can damage the alliance (Hussong and Micucci, 2020).

Integrating humor into coaching, aligned with established techniques like Cognitive Behavioral Coaching (CBC), offers valuable benefits. Lai and Palmer (2019) highlighted CBC’s widespread use, employing cognitive behavioral strategies to help clients achieve realistic goals and navigate change. Ellis and Bernard (1985), within the well-researched cognitive-behavioral framework, used humor to transform dysfunctional beliefs in clients. Transferring these advantages to coaching, humor aids in accepting mistakes, leads to better solutions, and facilitates the abandonment of old habits. Additionally, humor serves as a distraction from self-deprecating thoughts and provides didactic opportunities to break intervention monotony.

A less explored facet of humor is the phenomenon of failed humor, wherein humor falls short of achieving its intended effects. In the case of leaders, this refers to instances where a leader’s humor fails to amuse their followers. Failed humor can also undermine the relationship as is known from organizational literature (Pundt et al., 2022). To enhance coaches’ understanding of navigating failed humor they must be careful if mental conditions like autism or borderline are suspected, aware of the cultural background, and avoid forced humor (Hussong and Micucci, 2020). This contributes to greater sensitivity, and authenticity and fosters a more supportive coaching relationship when using humor. Applying WA insights from adjacent fields to coaching is promising, given the shorter duration of coaching engagements in comparison to therapy and counseling (Grant and Green, 2018). This is because there is less time to build rapport and trust, and humor can be a helpful tool for rapidly creating a connection with clients.

The Broaden and Build theory aligns with coaching’s context, asserting that positive emotions foster resilience and creativity (Fredrickson, 2001). This corresponds with our findings of humor affecting the professional dialogue by providing an improvement of cognitive abilities, more creativity (Hussong and Micucci, 2020; Kneisel et al., 2022), and a variety of responses (Kneisel et al., 2022), which challenge and shift beliefs (Gibson and Tantam, 2018; Kneisel et al., 2022). According to the Broaden and Build theory, positive emotions can expand thoughts and actions, creating lasting personal resources—physical, social, and psychological. This process encourages experimentation, risk-taking, and innovation leading to novel strategies for effective adaptation (Fredrickson and Joiner, 2002). The alignment of provocative coaching with the Broaden and Build theory is based on the intentional use of humor. This positive emotion fosters flexible thinking, risk-taking, adaptation, and innovation, all pivotal to effective coaching (Farrelly and Lynch, 1987; Vendl, 2017). These results may be especially transferrable to positive psychology coaching (PPC) as it is a popular paradigm for coaches.

Our findings hold relevance within executive coaching and the current landscape. de Vries and Rook (2018) highlight that executive coaches often have to deal with the dysfunctional behavior of clients, unaccustomed to receiving feedback. They also note that there is a growing concern about narcissistic leadership and its impact on individuals, teams, and organizations like lack of empathy, toxic work environments, high turnover, and ethical concerns. Our study identifies humor as a tool for coaches to address challenging clients (Schapiro-Halberstam et al., 2020; Graßmann et al., 2021). In our opinion, humor can be used to encounter these clients in a way less threatening than other methods, such as confrontation. Theory of Kohut (1978) states that maladjusted behaviors like narcissism, grandiosity, and entitlement, can potentially transform through the use of humor. Humor can create a safe space for clients to explore guilt- and shame-ridden experiences, even if the personality of the client displays narcissism, grandiosity, and “macho” behavior (Lachmann, 2003; Fabian, 2011). We found instances where aggressive humor empowered a therapist to engage with a difficult client (Schapiro-Halberstam et al., 2020) and “softening the blow to the ego” (Gibson and Tantam, 2018, p. 70) indicating that it could have a positive effect on clients with self-esteem. Considering humor as a character strength (Edwards and Martin, 2014; Wellenzohn et al., 2018), researching its role in assisting executive coaches with challenging clients presents a promising avenue. Our analysis suggests confrontational humor’s potential benefit for maladjusted behaviors, despite the assertions of Schneider et al. (2018) that therapeutic methods like Provocative and Rational Emotional Therapy may be less beneficial.

Apart from the above-mentioned issues, future research should also reflect on the various outcomes of humor use in coaching and categorize them to understand the field and establish theoretical frameworks underlying them. No broad classification framework for outcomes of humor use exists, but in all our studies there are a wide variety of positive and negative outcomes, all named in a slightly different way. Potential outcomes for example, enhance comfort for the client; ease the process; regulate emotion; lighten the atmosphere, focus the process; humanize the therapists; function as an avoidance strategy for the client; increase satisfaction with the practitioner; offend the client; model behavior and fosters in-depth exploration. As such, research can be done to identify all the positive and negative outcomes, cluster them into categories, and determine if the outcome is targeted at the client, practitioner, or the process. This could be connected to a coaching model (Theeboom et al., 2017) to identify in which phase of the coaching process its use would be appropriate.

More research is needed to investigate the actual casual links between humor use and outcome, also in the coaching field. A demonstrated high correlation (Panichelli et al., 2018) is no guarantee that causality is being found and data were retrospectively analyzed, introducing the potential for reporting bias and uncontrolled confounding factors, thereby posing a risk to the validity of the findings. As this work has shown eight studies relied on interviews of practitioners, although recommended to grasp the influence of humor as a social process, give way to biased information. Randomized controlled trials in which a subsample receives an intervention that promotes the WA for example can be used to assess the potential benefits of humor on WA. Such studies as well as other designs would contribute to the existing knowledge of this area and the coaching literature.

A promising domain that is gathering increased attention is the realm of virtual reality agents employed for personal development. As actual humans strive to enhance communication through social influences, humor emerges as a potent strategy, aligning with the positive effects expounded in this study. Ongoing research delves into the utilization of humor in virtual humans in counseling interviews (Kang et al., 2017) or for augmenting the social power strategies of robots (Hashemian et al., 2023). The findings of Kang et al. (2017) demonstrate that humor positively affects user responses (self-disclosure) and perceptions of a virtual counselor. Given that coaching is widely used in organizations, research into making virtual reality coaching effective could save considerable costs. Additionally, a comprehensive examination of human-agent interaction could furnish deeper insights into the nuances of humor recognition, comprehension, and appreciation. Consequently, such insights would significantly contribute to the judicious application of humor in professional dialogues, including coaching.

We conclude with an insight concerning the HRD literature. Coaching constitutes a core component of HRD, but we found no literature addressing the impact of humor in professional dialogue within this field. Apart from references suggesting that professional coaches may incorporate humor, as discussed in our introduction (Joo, 2005), no further elaboration was identified. HRD literature stands to benefit from the insights gleaned from our work, particularly within the realm of professional practice in coaching, mentoring, or leadership.

### 4.3 Practical implications

Coach education programs should follow an evidence-based approach. Coaches share a lack of training in the area of humor, which also leads to not using it. There is not much research on the topic of how practitioners could learn to use humor. Franzini (2001) who studied the topic suggests that humor is often discouraged in counseling or therapy, although it can be a useful tool for counselors. Valentine and Gabbard (2014) challenge the traditional view that humor in professional dialogue is risky and should be avoided. Their implications for training are the following: to teach humor effectively requires personalized attention in training sessions and supervision. The therapist needs to have a natural inclination for humor, the ability to mentalize (understanding feelings and thoughts), and a genuine interest in using humor in sessions. Furthermore, while some trainees may not have a natural aptitude for humor, all can benefit from exploring how to respond to patient-initiated humor (Valentine and Gabbard, 2014). This corresponds with Briggs and Owen (2022) argue that humor can benefit both clients and practitioners; at least a discussion about the role of humor during training courses should be added. So that therapists could learn to be more authentic and feel free to reveal themselves in contrast to being a distant analyst (Briggs and Owen, 2022). Moreover, to diminish previously mentioned risks (belittling or making fun of the client), it might be mandatory for practitioners to be trained to improve their sense of humor and integrate humor into their practice (Dionigi and Canestrari, 2018). Also, in our review, there is some proof, that practitioners should know how clients use humor, for example as a way of coping and not revealing important (painful) topics (Hussong and Micucci, 2020). Using humor can be an effective tool for practitioners, as long as they are used with skill and sensitivity (Gelkopf and Kreitler, 1996). So, we argue, based on our findings, that it is time to educate coaches (and other practitioners) on how to use this skill. This trend is consistent across more study fields like in workplace literature (Romero and Cruthirds, 2017; Rosenberg et al., 2021; Li et al., 2023).

Following our results coaches should be aware and learn when to use humor, how to target it, and in which phase to use it. Supervision is a perfect way to help coaches with this aim, as supervision is a way to augment coaches’ skills and efficacy (de Haan, 2016; Joseph, 2016; Graßmann et al., 2021). This could help coaches to broaden their repertoire of types of clients. We suspect that when a coach can extend the range of clients this will also add up to their effectiveness and self-consciousness. Coaches who, for example, are coaching on the executive level, may have more tools and therefore be more effective when they learn to use humor properly.

### 4.4 Limitations

Our review is not exempt from some limitations. The conclusions that we draw come from adjacent literature. Although similarities exist between mentoring, counseling, psychotherapy, and coaching, there are also significant differences in the type, intensity, context, foci, and duration of the alliances. Although all the studies were about humor some had a slight emphasis on laughter. Given the array of different dialogues used, coaching, mentoring, therapy, and counseling, our study opens the room for more research centered in the coaching practice. In our review, some elements related to humor like gender or culture have not been considered in the analysis. Only one study elaborated on humor use as a tool for helping to overcome the female stereotype role expectation with her client (Schapiro-Halberstam et al., 2020). Further studies are encouraged to include these variables, which have also relevance in coaching and humor understanding acting as mediators or moderators of this relationship (Evans et al., 2019; Rosenberg et al., 2021).

The number of participants in the selected studies varies between 1 and 108. Studies 6–13 had fewer than 10 participants. We added two case studies to get more in-depth insight into how humor functions, studying only one practitioner and one client but questions can be raised about whether results have validity. Both types of professionals and the mental health states of clients are heterogeneous in the studies reviewed. This implies that our insights may show some nuances according to the type of professional context and mental health issues.

### 4.5 Conclusion

Our systematic review delves into the multifaceted role of humor within professional dialogues. Despite limitations on data of ethnicity and gender as well as the heterogeneity of the studies, our study contributes to a deeper understanding of humor’s dynamic role in coaching. By analyzing 13 studies, alongside prior humor and coaching reviews, we address the paucity of comprehensive evidence supporting the positive impacts of humor, bridging our findings to the coaching domain. Our findings underscore the correlative relationship between humor use and favorable outcomes, spanning benefits for clients’ mental well-being, and organizational aspects like job satisfaction. Notably, humor proves to be instrumental in establishing and strengthening the working alliance (WA), challenging conventional beliefs that humor should be employed only after alliance establishment. Aggressive humor emerges as a valuable tool for both clients and practitioners, allowing the expression of challenging emotions and managing interactions. Looking forward, our recommendations advocate for qualitative and mixed methods research designs, exploring humor’s previously uncharted outcomes. By enhancing coach education programs with insights into how humor operates, coaches could mitigate the risks humor carries, namely offending the client or inappropriate use. Additionally, the emergence of virtual reality agents in personal development presents a promising avenue for further research. As the coaching landscape increasingly focuses on trust-building and positive emotions, our findings offer valuable insights for enhancing coaching efficacy. This, in turn, should encourage practitioners and researchers to explore humor’s potential for fostering meaningful connections and positive outcomes.

## Author contributions

AV: Writing – original draft. CA-A: Methodology, Supervision, Writing – review & editing. ME: Conceptualization, Methodology, Supervision, Writing – review & editing.
